# Effects of Herbal Therapy on Intestinal Microbiota and Serum Metabolomics in Different Rat Models of Mongolian Medicine

**DOI:** 10.1155/2022/7255780

**Published:** 2022-05-30

**Authors:** Guniang Jiu, Riao Dao, Dongxing Wu, Wang Hung, Haburi Jin, Li Li, Xiquan Fu, Chula Sa, Eerdunchaolu

**Affiliations:** ^1^Mongolian Medical College, Inner Mongolia Minzu University, Tongliao 028000, Inner Mongolia, China; ^2^Affiliated Hospital of Inner Mongolia Minzu University, Tongliao 028000, Inner Mongolia, China; ^3^Department of Psychosomatic Medicine, International Mongolian Medical Hospital of Inner Mongolia Autonomous Region, Hohhot 010000, Inner Mongolia, China; ^4^Affiliated Hospital of Inner Mongolia Minzu University, Rehabilitation Hospital, Tongliao 028000, Inner Mongolia, China; ^5^Second Department of Encephalopathy, Affiliated Hospital of Inner Mongolia Minzu University, Tongliao 028000, Inner Mongolia, China

## Abstract

**Objective:**

Heyi disease, Xila disease, and Badagan disease are three common diseases in Mongolian medicine. The changes in intestinal microbiota may be associated with the occurrence, development, and treatment of these diseases. This study aimed to investigate the effects of herbal treatment on intestinal microbiota and serum metabolites in rats with these three diseases.

**Methods:**

Firstly, Heyi, Xila, and Badagan disease model rats were established by environmental, diet, and drug intervention. Then, 16S rRNA gene sequencing and metabolomics analysis were used to analyze the changes in intestinal microbiota and serum metabolites after treatment. PICRUSt analysis was applied to predict the potential functions of intestinal microbiota, and OPLS-DA multivariate model was applied to screen differential serum metabolites.

**Results:**

16S rRNA gene sequencing showed that herbal treatment significantly increased the species diversity and changed the composition of intestinal microbiota in Heyi disease and Xila disease rats. After treatment, there were 10, 9, and 3 bacterial biomarkers that were increased in Heyi, Xila, and Badagan disease rats, respectively. In the Heyi disease model, treatment resulted in 45 differential serum metabolites, involving 4 pathways. In the Badagan disease model, treatment resulted in 62 differential serum metabolites, involving 4 pathways. However, there was no significant difference in serum metabolites between TreatB and ConB in the Xila disease model.

**Conclusions:**

Herbal treatment significantly changed the intestinal microbiota and serum metabolites of rats with three Mongolian medicine diseases.

## 1. Introduction

Mongolian medicine (MGM) is an important branch of traditional medicine in China. MGM believes that the balance among the three life-sustaining principles (including Heyi, Xila, and Badagan) is the basis of human health. Once this balance is broken by various pathogenic factors, it will lead to disease [1]. According to the “Four-Part Medicine Classics,” [2] these three principles were endowed with different attributes. Heyi belonged to Qi and was characterized by lightness and movement. Therefore, Heyi disease was considered as a Qi-related disease, and the symptoms of Heyi disease include sighing, upset, insomnia, dyspepsia, bloating, and constipation. Xila belonged to fire, and was characterized by heat, which was considered as a heat-related disease, and the symptoms of Xila disease include fever, headache, thirst, and excessive sweating. Badagan belonged to water, and was characterized by cold, which was considered as a cold-related disease, and the symptoms of Badagan disease include slow reaction, dyspepsia, loss of appetite, and vomiting. Abnormal climate change, chronic lack of nutrition, coarse food, and mental stimulation are the causes of these diseases, but the underlying mechanisms remain unclear. Studies have shown that intestinal microbiota disorders linked poor eating habits and unhealthy lifestyles with diseases [3]. Whether the intestinal microbiota contributed to the pathogenesis and treatment of these three diseases remains to be further studied.

Currently, the intestinal microbiota is considered to be a new complex organ composed of 10^13^ to 10^14^ bacteria, which is more than 10 times the total number of human cells [4]. Intestinal microbiota is one of the important factors of intestinal microenvironment homeostasis, and its changes may affect immune and metabolic functions, leading to various autoimmune and intestinal diseases [5]. Studies have shown that several diseases, such as Type 2 diabetes mellitus, Parkinson's disease, Alzheimer's disease, and malignant tumors, were closely related to intestinal microecological disorders [6, 7]. The regulation of intestinal microbiota was also considered as a treatment for some diseases, since increasing the proportion of beneficial bacteria and restoring the intestinal barrier could promote the health of the host and reduce the risk of disease [8]. For example, Liu et al. showed that *Pulsatilla chinensis* Saponins significantly improved dextran sulfate sodium-induced ulcerative colitis and reduced inflammatory response by regulating intestinal microbiota composition and biodiversity [5]. Another study showed that soluble dietary fiber protected the intestinal mucosal barrier by improving the intestinal microbiota in septic mouse [9]. Our previous studies have shown that the intestinal microbiota of rats has changed significantly after suffered from these three diseases [10]. Further study on the relationship between intestinal microbiota and disease may bring new insights into the treatment.

Metabolomics is a comprehensive analytical approach for the study of biological efficacy and mainly used to evaluate the effects of disease status or drug treatment on endogenous metabolites such as amino acids, fatty acids, lipids, and peptides [11]. At present, the metabolomic investigation has been widely used to evaluate the biological efficacy and underlying mechanism of traditional Chinese medicine [12]. A recent metabolomic study revealed that 23 biomarkers were identified in rat fatty liver after treatment with Qushi Huayu decoction [13]. Meanwhile, a metabolomic approach based on LC-Q-TOF/MS identified 27 biomarkers in the serum of myocardial infarction rats, which involved in 4 main pathological processes including oxidative injury, energy metabolism dysfunction, amino acid metabolism dysfunction, and inflammation [14]. However, the characteristics of serum metabolites after treatment of Heyi, Xila, and Badagan disease have not been revealed. Hence, the integration of metabolomics and intestinal microbiota analysis would help us better understand disease and treatment in Mongolian medicine. *Ferula sinkiangensis* K. M. Shen (FS), *Lomatogonium carinthiacum* (LC), and *Punica granatum* L. (PG) were commonly used herbs in MGM, and they have good therapeutic effects on Heyi disease, Xila disease, and Badagan disease, respectively. FS is a medicinal plant of the family *Umbelliferae*, mainly distributed in Xinjiang of China, and FS has been recorded in a variety of traditional medicine. The FS was widely used in stomach disease and anticancer [15, 16]. LC is a member of the family *Gentianaceae* and is mainly distributed in Inner Mongolia, Shanxi, and Xinjiang of China, which was commonly used to treat influenza, typhoid, liver disease, and jaundice in MGM [17]. PG is a medicinal and edible plant, derived from the family *Punicaceae*, which is a popular healthy fruit worldwide [18]. As an MGM herb, PG was considered to have the effect of eliminating food and diarrhea and was often used to treat dyspepsia. Therefore, these three herbs were used to treat rats with MGM disease in this study.

This study aimed to investigate the therapeutic effects of different Mongolian herbs on three diseases by 16S rRNA gene sequencing and metabolomics analysis. These results will also provide a deeper understanding of the relationship between intestinal microbiota and MGM disease treatment.

## 2. Methods and Materials

### 2.1. Materials

Black tea (Fujian Anxi County Huayuan Tea Industry Co., Ltd., Wuyi Mountain, China, production standard: GB/T13738.3), buckwheat (Inner Mongolia Qinggu Xinhe Agricultural Science and Technology Co., Ltd., China, batch number: SC10115059900036), Mongolian medicine Gaburi (*Dryobalanops aromatica* Gaertner f., Preparation Center of Inner Mongolia University for Nationalities, batch number: 20190512), liquor (Inner Mongolia Taifusi Qi Grassland Brewing Co., Ltd., China, batch number: SC11515252800086), yellow rice (Yuxian Xifu Agricultural Products Trading Co., Ltd., China, batch number: SC10113072600272), black pepper (Henan Gujin Food Therapy Technology Co., Ltd., China, batch number: SC10341172900240), sunflower oil (Jiage Food Co., Ltd., China, batch number: SC10215082200114), *Taraxacum mongolicum* Hand.-Mazz. (Preparation Center of Inner Mongolia University for Nationalities, batch number: 20190615), FS (Preparation Center of Inner Mongolia University for Nationalities, batch number: 20190513), LC (Preparation Center of Inner Mongolia University for Nationalities, batch number: 20190721), and PG (Preparation Center of Inner Mongolia University for Nationalities, batch number: 20190622).

### 2.2. Herbal Medicine Preparation

FS: Briefly, 13 g FS was weighed and boiled with ultrapure water for twice, 50 mL each time. After filtration, the filtrate was combined and concentrated to 10 mL, and the concentration was 1.3 g/mL. The rats were given 1 mL intragastric administration of 100 g body weight at a dose of 1.3 g/kg (g/kg, ratio of crude drug dosage to rat body weight). The preparation of LC and PG was the same as that of FS.

### 2.3. Animals and Treatments

Healthy male SD rats (*n* = 60, weight: 180 ± 20 g) were provided by Liaoning Changsheng Biotechnology Co., Ltd. (Liaoning, China). The rats were kept in standard conditions with 12 h light/dark cycles, temperature 20–23°C, and humidity 40 ± 5%, and the rats had free access to food and water. After one week of adaptive feeding, rats were then grouped randomly into six groups (*n* = 10 per group): ConA (Heyi rat model), TreatA (Heyi rats treated with FS, 1.3 g/kg), ConB (Xila rat model), TreatB (Xila rats treated with LC, 1.3 g/kg), ConC (Badagan rat model), and TreatC (Badagan rats treated with PG, 1.3 g/kg).

After the rat models of the three diseases were established, the rats were treated with the corresponding herbs. The therapeutic dose was converted according to the human dose used in MGM. MGM recommended that the daily dosage of these three herbs should not exceed 15 g. In consideration of the average weight of an adult was 70 kg, the adult oral dose was 0.21 g/kg/d. After multiplied by the conversion coefficient of body surface area between human and rat (6.3), the daily oral dose of rats was about 1.30 g/kg. The treatments were administered intragastrically once daily for 14 days.

### 2.4. Three Rat Models of MGM Diseases

Traditional medicine believed that Heyi, Xila, and Badagan diseases were caused by abnormal climate change, chronic lack of nutrition, coarse food, and mental stimulation. When the rats showed the characteristics of Heyi, Xila, and Badagan described in “Four-Part Medicine Classics,” it was considered that the model was successfully constructed [2]. Therefore, we conducted environmental, diet, and drug interventions on the model group [10]. All experimental protocols were approved by the Medical Ethics Committee of the Affiliated Hospital of Inner Mongolia University for the Nationalities (ethic code: NM-LL-2019-12-06-01). The following three MGM disease models (MGM-DM) were established as follows:

Heyi rat model (ConA): the rat's daily drinking water was replaced by black tea (5 g/100 mL); buckwheat (8.5 g/day) was added to the diet; Gaburi solution (1 mL/100 g/day) was administered, and 0.1 mL tail vein bloodletting was performed on the rats at 5 pm every two days. In addition, rats were exposed to the continuous cat audio at 70 decibels. These interventions continued for 31 days. After modeling, the rats with Heyi disease showed significantly decreased activity, withered hair, listlessness, and slow response.

Xila rat model (ConB): rats were kept at 29 ± 2°C, and rat chow was replaced with yellow rice (15 g/day). 0.7 g/kg sunflower oil was gavaged at 6 am, and 0.7 g/kg pepper was gavaged at 12 noon every day. In addition, rats were given 1 mL liqueur once every other day. These interventions continued for 21 days. After modeling, Xila disease rats showed drowsiness, dull hair, significantly reduced diet, soft stool, and yellow urine.

Badagan rat model (ConC): rats were reared at 60 ± 5% humidity, and rat chow was replaced with lard and wheat flour (ratio 1 : 4). The rats were gavaged 4 mL *Taraxacum mongolicum* Hand-Mazz. (200% decoction). These interventions continued for 49 days. After modeling, Badagan disease rats showed dirty hair, listlessness, curling, loose stools, diarrhea, and low body temperature.

### 2.5. Fecal Sample Collection and DNA Extraction

The collected fresh fecal samples of all rats were stored in sterile EP tubes and frozen at −80. The E.Z.N.A. fecal DNA Kit (omega bio TEK, Norcross, GA, United States) was used to extract DNA from fecal samples according to the manufacturer's instructions. Then, the purity and concentration of DNA were determined by NanoDrop 2000 spectrophotometer (Thermo Fisher Scientific, United States), and DNA quality was detected by 1% agarose gel electrophoresis.

### 2.6. 16S rRNA Gene Sequencing and Analysis

For analysis of the intestinal microbiota, extracted DNA was sequenced by the Illumina HiSeq sequencing platform (Illumina, USA), and the V3-V4 hypervariable region of the 16S rRNA gene was targeted with the primers 341F (5′-CCTAYGGG- RBGCASCAG-3′) and 806R (5′-GGACTACNNGGGTATCTAAT-3′). Sequence assembly, quality control, and clustering were performed using FLASH (version 1.2.11) and USEARCH (version 10.0).

### 2.7. Serum Sample Preparation for HPLC-MS/MS

At the end of treatment, rats were fasted for 12 hours and euthanized under anesthesia. Then, blood was collected from the abdominal aorta, and the collected blood sample was centrifuged at 4500 rpm for 10 min to separate serum. The 200 *μ*L serum was mixed with the precooled acetonitrile at a volume ratio of 1 : 3 and then vortexed for 60 s. The mixed sample was kept at −20°C for 30 min to allow the compounds in the samples to be fully extracted. Next, the mixed sample was centrifuged at 4°C for 14000 g for 15 min, and the supernatant was transferred to a new EP tube for concentration until the solvent was completely volatilized. Finally, the sample was redissolved with a mixture of ammonium acetate and acetonitrile at a volume ratio of 1 : 1. After centrifugation at 14000 g for 10 min, the supernatant was analyzed by HPLC-MS/MS.

### 2.8. Serum Metabolomic Analysis

Serum metabolites were analyzed by Thermo Scientific Q Exactive mass spectrometer. The Accucore Hilic C18 column (100 × 2.1 mm, 2.6 *μ*m) was used to perform the chromatographic separation, and the column temperature was kept at 35°C. The mobile phase consisted of 10 mM ammonium acetate (*A*) and acetonitrile/10 mM ammonium acetate (9 : 1) (*B*). The flow rate was 0.35 mL/min, and injection volume was 2 *μ*L. The gradient elution program was as follows: 0∼1 min, 100% *A*; 1∼9 min, 0%∼100% *B*; 9∼12 min, 100% *B*; and 12.1∼15 min, 100% *A*. The MS analysis was worked using full scan mode, and the mass range was recorded from *m*/*z* 70 to 1050 both in positive and negative mode. The parent ions with TOP10 ionic strength were selected for secondary MS identification. HCD method was used to fragment the parent ion, and the secondary MS sequence was determined to generate the original file of MS detection. Then, Compound Discover V3.0 software was applied to extract, control, and normalize the original data. Metabolites were identified by mzCloud database (https://www.mzcloud.org/) and ChemSpider database (https://www.chemspider.com/). The compound spectrograms in mzCloud database were obtained through the collection of standard substances. And the spectrogram library contained the secondary or multilevel MS spectrograms generated by different collision energies of CID (collision-induced dissociation) and HCD (higher energy collision-induced dissociation) fragmentation modes. Moreover, the structure information of fragment ions was annotated in the MS spectrogram to facilitate the structure identification of unknown compounds. The ChemSpider database contained more than 30 million structures, providing more detailed information about compounds.

Orthogonal partial least square discriminate analysis (OPLS-DA) was performed by SIMCA-P software. OPLS analysis generated VIP-plot (VIP >1) to select different variables as potential markers. The differential metabolites were screened with VIP >1 and *p* < 0.05.

### 2.9. Statistical Analysis

Wilcoxon signed rank-sum test was used to analyze the alpha diversity index and bacterial community, and statistical significance was defined at *p* < 0.05. PERMANOVA analysis was used to test the differences between groups. Kruskal–Wallis sum-rank test was applied to analyze differential biomarker in line discriminant analysis (LDA) effect size (LEfSe) analysis, with | LDA score | > 3 and *p* < 0.05 as screening threshold.

## 3. Results

### 3.1. Changes in Intestinal Microbiota Diversity in Different MGM-DM Rats after Treatment

Changes in intestinal microbiota were thought to be involved in the occurrence, development, and treatment of diseases [5]. To determine whether the intestinal microbiota of rats changed after treatment, 16S rRNA gene sequencing analysis was performed. Alpha diversity contains four indices ACE, Chao1, Shannon, and Simpson, which is an analysis of species diversity in a single sample. ACE and Chao1 reflect the community richness of species, while Shannon and Simpson represent microbial diversity [5]. As listed in Table 1, there were no significant differences in ACE and Chao 1 between the three MGM-DM groups and the corresponding treatment groups (*p* > 0.05). Among the three treatment groups, the Shannon value of TreatA and TreatB was higher than that of the corresponding MGM-DM group, and only the Simpson value of TreatA decreased significantly.

**Figure 1:**
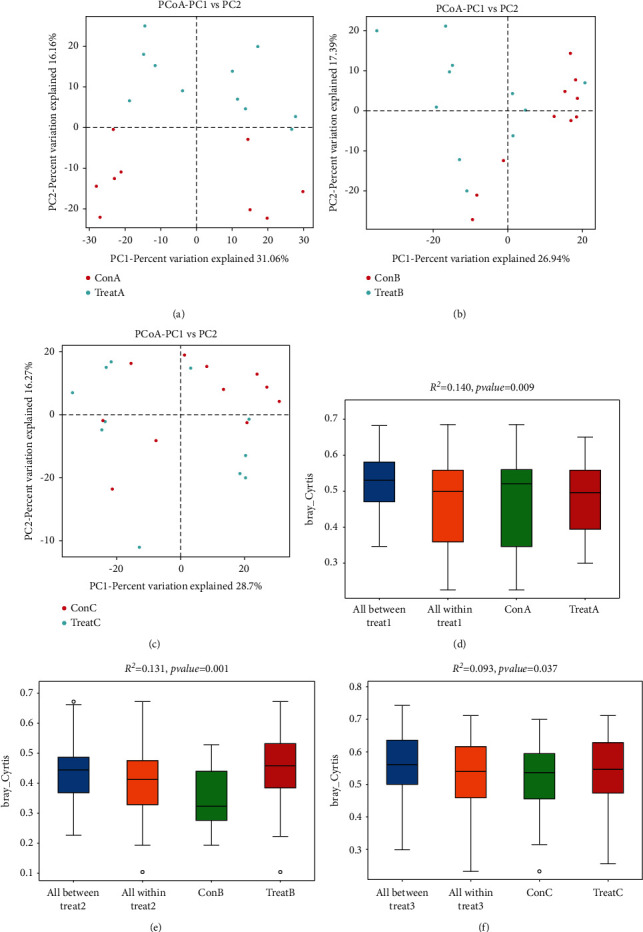
Beta diversity analysis of intestinal microbial community. (a)–(c) PCoA analysis of variation between the bacterial communities presenting in all groups. (a) Heyi disease model, (b) Xila disease model, and (c) Badagan disease model. Each data point represented an individual sample. (d)–(f) PERMANOVA analysis of three MGM-DM groups and treatment groups. *Y*-axis: Bray Curtis distance. *p* < 0.05 was considered as significant difference.

We also applied principal coordinates analysis (PCoA) and PERMANOVA analysis to evaluate the beta diversity of all samples. The results showed that Heyi and Xila disease model groups and their treatment groups could be clearly distinguished in the PCoA diagram (Figures 1(a) and 1(c)), while the differences between the Badagan disease model group and the treatment group were not significant (Figure 1(b)). Further analysis by PERMANOVA showed significant differences between all MGM-DM groups and the corresponding treatment groups (*p* < 0.05, Figures 1(d)–1(f)). Such data indicated that the treatment did not cause a significant change in the abundance of intestinal microbial species, whereas the diversity of intestinal microbial species was increased both in Heyi disease rats and Xila disease rats after treatment.

### 3.2. Alterations of Intestinal Microbiota Composition in Different MGM-DM Rats after Treatment

**Figure 2:**
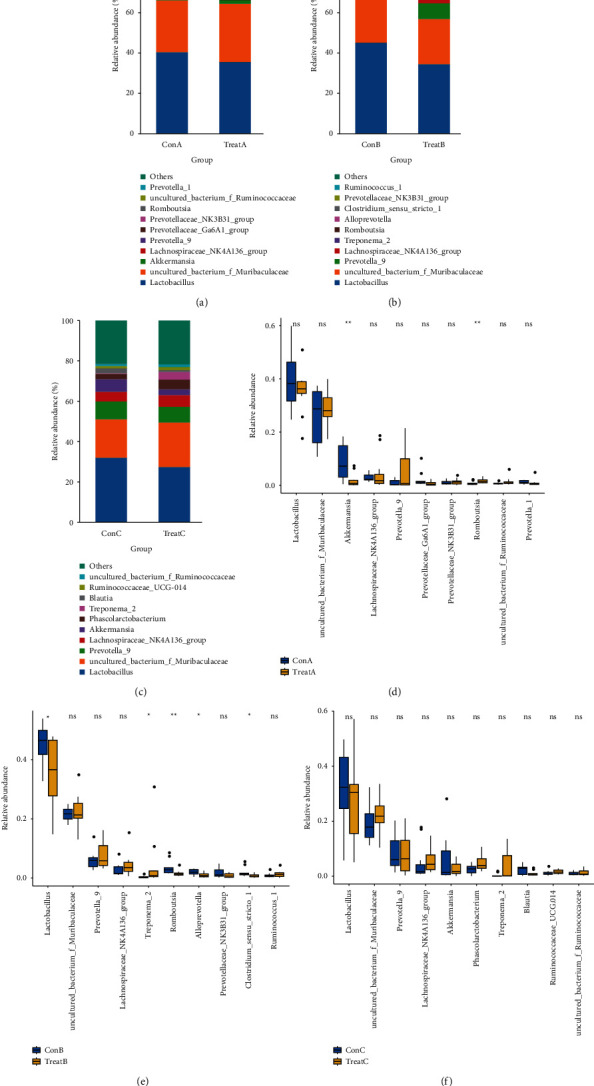
Changes in composition of Top10 intestinal microbiota at genus levels in each group. (a)–(c) Intestinal microbial composition at genus level in rats. (a) Heyi disease model, (b) Xila disease model, and (c) Badagan disease model. (d)–(f) intestinal microbial abundance at genus level in rats. (d) Heyi disease model, (e) Xila disease model, and (f) Badagan disease model.

Next, we analyzed the alterations of intestinal microbiota composition of rats after treatment. At the genus level, *Lactobacillus* and *uncultured_bacterium_f_ Muribaculaceae* accounted for the largest proportion in all groups (Figures 2(a)–2(c)). In Heyi disease model, compared with ConA group, the abundance of *Akkermansia* in TreatA group was significantly decreased, while the Romboutsia was significantly increased (*p* value < 0.05, Figure 2(d)). In Xila disease model, compared with ConB group, the abundance of Lactobacillus, Romboutsia, Alloprevotella, and Clostridium_sensu_stricto_1 in TreatB group was significantly decreased, while the Treponema_2 was opposite (*p* value < 0.05, Figure 2(e)). However, there was no significant difference in the abundance of the top10 bacteria at the genus level between TreatC group and ConC group (Figure 2(f)).

**Figure 3:**
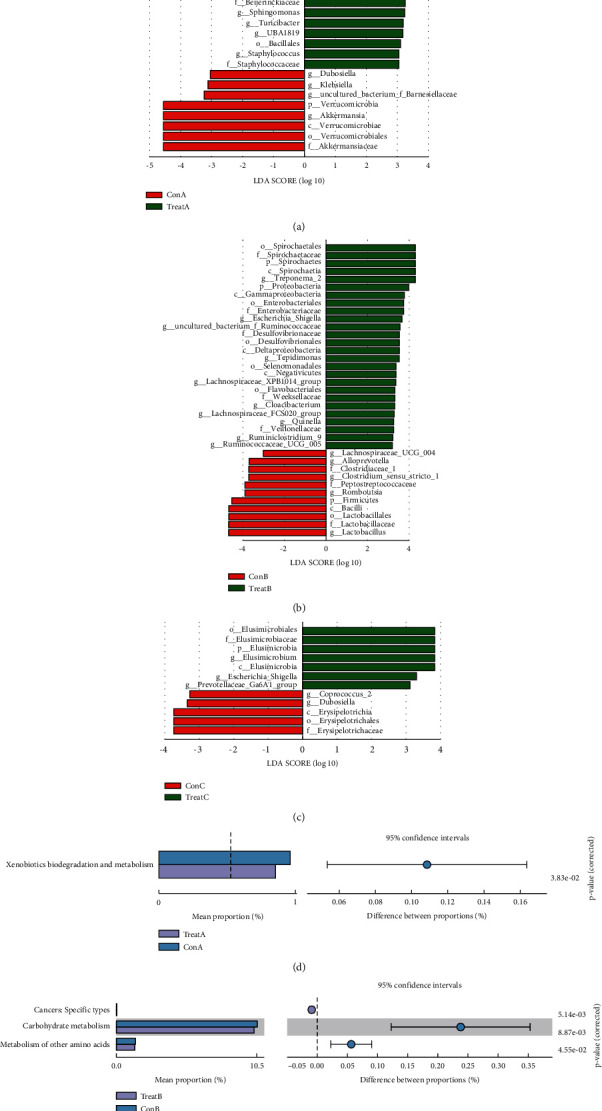
LEfSe analysis and KEGG function prediction. (a) The significantly differential biomarkers between the Heyi rat model and treatment group based on LEfSe analysis; (b) the significantly differential biomarkers between the Xila rat model and treatment group based on LEfSe analysis; (c) the significantly differential biomarkers between the Badagan rat model and treatment group based on LEfSe analysis; (d) differential analysis of KEGG function prediction between Heyi rat model and treatment group; (e) differential analysis of KEGG function prediction between Xila rat model and treatment group.

To identify the specific intestinal bacterial biomarkers (BBM) at the genus level, LEfSe analysis was used. TreatA group had 10 groups of BBM with significantly higher abundance than that of ConA group, including *Bacillales*, *Lachnospiraceae_XPB1014_group*, *Peptostreptococcaceae*, *UBA1819*, *Erysipelotrichia*, *Beijerinckiaceae*, *Sphingomonas*, *Desulfovibrio*, *Tepidimonas*, *Moraxellaceae* (Figure 3(a)). TreatB group had 9 groups of BBM with significantly higher abundance than that in ConB group, including *Flavobacteriales, Lachnospiraceae_FCS020_group*, *Lachnospiraceae_XPB1014_group*, *Ruminiclostridium_9*, *Ruminococcaceae_UCG_005*, *uncultured_bacterium_f_Ruminococcaceae*, *Negativicutes*, *Proteobacteria*, *Spirochaetes* (Figure 3(b)). TreatC group had 3 groups of BBM with significantly higher abundance than those of ConC group including *Prevotellaceae_Ga6A1_group*, *Elusimicrobia* (from phylum to genus: *Elusimicrobia*, *Elusimicrobiales*, *Elusimicrobiaceae*, *Elusimicrobium*), *Escherichia_Shigella* (Figure 3(c)).

### 3.3. Prediction of Intestinal Microbial Function

To better understand the functional changes associated with perturbation in microbial composition, PICRUSt analysis was applied to predict the potential functions of intestinal microbiota. Compared with ConA, the abundance of “xenobiotics biodegradation and metabolism” pathway in TreatA was significantly increased (Figure 3(d)). Compared with ConB, the abundance of “cancers: specific types” pathway was significantly decreased, while the abundance of “carbohydrate metabolism” pathway and “metabolism of other amino acids” pathway was significantly increased in TreatB (Figure 3(e)). And there was no significant difference between TreatC and ConC in each pathway.

### 3.4. Identification of Serum Metabolic Markers

**Figure 4:**
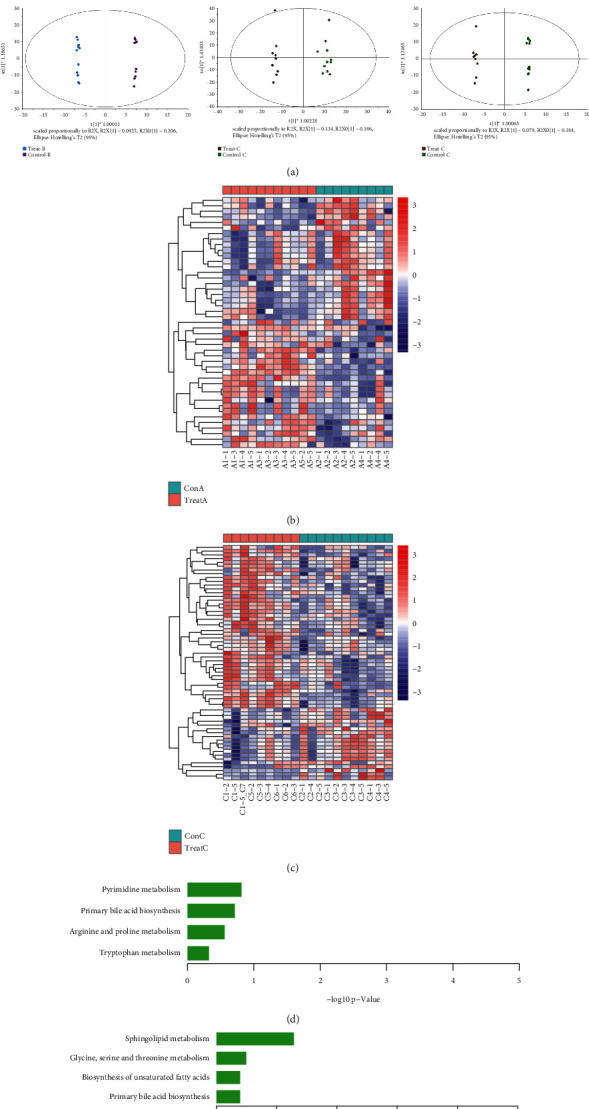
Serum metabolic profiling. (a) The OPLS-DA score plot of three MGM-DM groups and corresponding treatment groups on positive ion and negative ion modes; (b) heat map of metabolic levels of differential metabolites between Heyi rat model and treatment group; (c) heat map of metabolic levels of differential metabolites between Badagan rat model and treatment group; (d) KEGG enrichment analysis of differential metabolites in Heyi rat model and treatment group; (e) KEGG enrichment analysis of differential metabolites in Badagan rat model and treatment group.

Here, serum metabolic profiles of all rats were obtained from HPLC-MS/MS in positive and negative modes. In addition, we used OPLS-DA analysis to evaluate whether there were differences between the disease group and the treatment group. As shown in Figure 4(a), the metabolic profiles of the three disease groups and their corresponding treatment groups were significantly separated, indicating that the endogenous metabolites of rats changed significantly after treatment. Next, we screened differential metabolites in the OPLS-DA analysis with VIP >1 and *p* < 0.05 as the threshold. Compared with ConA group, 26 differential metabolites under positive ion mode and 19 different metabolites under negative ion mode were detected in TreatA group (Table S1), resulting in 45 differential metabolites involved in 4 metabolic pathways, including “primary bile acid biosynthesis,” “pyrimidine metabolism,” “arginine and proline metabolism,” and “tryptophan metabolism” (the metabolic heat map of 45 differential metabolites was shown in Figure 4(b)). Compared with ConC group, 40 differential metabolites under positive ion mode and 22 different metabolites under negative ion mode were detected in TreatC group (Table S1), resulting in 62 differential metabolites involved in 4 metabolic pathways, including “primary bile acid biosynthesis,” glycine, “serine and threonine metabolism,” “sphingolipid metabolism,” and “biosynthesis of unsaturated fatty acid” (the metabolic heat map of 62 differential metabolites was shown in Figure 4(c)).

## 4. Discussion

This study established three rat models with MGM disease and treated them with FR, LC, and PG herbs. Significant changes in intestinal microbiota and serum metabolites were detected in Heyi, Xila, and Badagan disease rats after treatment via 16S rRNA gene sequencing and metabolomics.

The poor dietary habits, harsh living environment, and negative mental stimulation could cause serious health problems [19–21]. Studies have shown that changes in intestinal microbiota link poor dietary habits to Alzheimer's disease [22]. Therefore, the intestinal microbiota may serve as a bridge between disease and risk factors and be considered as an effective way of treatment [7, 23]. In our previous studies, we found that the intestinal microbiota of rats with Heyi, Xila, and Badagan diseases changed significantly, accompanied by the decrease of beneficial bacteria and the increase of harmful bacteria [10]. In the present study, we observed no significant changes in the ACE index and Chao1 index in all groups, indicating that treatment did not change the abundance of intestinal microbiota in rats, but the diversity of intestinal microbial species of Heyi disease rats and Xila disease rats was significantly increased after treatment. Beta diversity analysis showed that there were significant differences between Heyi disease rats and FR treatment rats, as well as Xila disease rats and LC treatment rats, suggesting changes in microbial species composition, while these changes were not found in the Badagan rat model. In the Heyi disease model, the abundance of *Akkermansia* in rats was significantly decreased after treatment, while the *Romboutsia* was significantly increased. This suggested that *Akkermansia* and *Romboutsia* could be used as an indicator of Heyi disease treatment.

However, in previous studies, *Akkermansia* was considered beneficial because it could regulate the thickness of intestinal mucus and maintain the integrity of the he intestinal barrier [24]. In this study, we speculated that the decrease in *Akkermansia* abundance might be related to the treatment dose and treatment cycle. In the Xila disease model, compared with ConB group, the abundance of *Lactobacillus*, *Romboutsia*, Alloprevotella, and *Clostridium_sensu_stricto_1* in the TreatB group was significantly decreased, while the *Treponema_2* was opposite. We also found that there were 10, 9, and 3 BBMs that increased in Heyi, Xila, and Badagan disease rats after treatment, respectively. The disorder of intestinal microbiota led to the decline of intestinal barrier function and immunity, which further induced intestinal inflammation and caused a series of pathological reactions [25]. Therefore, regulating the dynamic balance of intestinal flora was conducive to health recovery. Moreover, changes in the KEGG pathway were compared before and after treatment. In the Heyi disease model, the abundance of “xenobiotics biodegradation and metabolism” pathway was significantly increased in the TreatA group. In the Xila disease model, the abundance of “cancers: specific types” pathway was significantly decreased, but the abundance of “carbohydrate metabolism” pathway and “metabolism of other amino acids” pathway was significantly increased in the TreatB group. We speculated that these pathways may play an important role in the treatment of three MGM-DM rats.

Furthermore, metabolomics revealed the effects of herbal treatments on serum metabolites. After treatment, a total of 45 different metabolites between TreatA and ConA were detected, involving four pathways: “primary bile acid biosynthesis,” “pyrimidine metabolism,” “arginine and proline metabolism,” and “tryptophan metabolism.” A total of 62 different metabolites between TreatC and ConC were detected, involving four pathways: “primary bile acid biosynthesis,” “glycine, serine and threonine metabolism,” “sphingolipid metabolism,” and “biosynthesis of unsaturated fatty acids.” We noticed that most of these metabolic pathways were related to amino acid metabolism and primary bile acid biosynthesis. Most of these metabolites have been reported to be involved in a variety of biological processes. For example, glycine was considered as a radical scavenger that prevented oxidative damage and apoptosis [26]. Tryptophan could promote intestinal immune defense [27]. Bile acids protected the integrity of intestinal barrier by inhibiting the overgrowth of intestinal bacteria. In turn, intestinal bacteria controlled the composition and pool size of circulating bile acids [28]. It is widely known that herbal medicine has the characteristics of multitarget, multipathway, and synergistic effect due to its complex chemical compounds [29]. For example, it was reported that PG contained a variety of polyphenols (such as gallic acid and ellagic acid), so PG extract had strong antibacterial, antioxidant, and free radical scavenging abilities [30]. Wu et al. found that the polysaccharide in PG could promote the proliferation of spleen lymphocytes and increase the expression of immunoglobulin in immunosuppressive model mice [31]. Ferulic acid, one of the main components in PG and FS, had a wide range of pharmacological activities, especially in inflammation, oxidative stress, and platelet aggregation [32, 33]. Jia et al. used HPLC-EIS-MS/MS method to identify 21 components in LC, including quercetin, luteolin, swertiamarin, gentiopicroside, and apigenin [34]. Recent studies have shown that quercetin could reduce the passive coping behavior induced by psychosocial stress via regulating the HPA axis and inhibiting brain oxidative stress and neuroinflammation [35]. In addition, luteolin has been shown to significantly alter the composition and richness of intestinal microbiota in rats with nonalcoholic fatty liver disease [36]. A large number of chemical components were considered as the key to the effectiveness of herbal medicine [37]. Therefore, the three herbs contain a variety of active ingredients, which were the material basis for the treatment of MGM disease rats, and these compounds caused the difference in metabolic level between the control group and the treatment group. In general, the metabolic pathways in MGM-DM rats were disordered, and FS, LC, and PG could effectively regulate the disordered metabolic pathways, and the mechanism might be related to the regulation of amino acid metabolism and primary bile acid synthesis.

Despite all this, there are some limitations to our study. We did not pay enough attention to the biochemical changes and pathological examination of rats before and after treatment. In addition, the influence of changes in intestinal microbiota on herbal therapy should be further explored.

## 5. Conclusion

In summary, 16S rRNA gene sequencing and metabonomics analysis were used to investigate the changes of intestinal microbiota and serum metabolites in rats of three Mongolian medicine disease models after treatment. Our results showed that intestinal microbiota and serum metabolites in treatment group rats were significantly different from that in model group rats. Our findings provide more clues for further study of Mongolian medicine.

## Figures and Tables

**Table 1:** Alpha diversity changes in rats with different diseases after treatment.

Alpha diversity index	Heyi disease rats			Xila disease rats			Badagan disease rats		
	ConA	TreatA	*p* value	ConB	TreatB	*p* value	ConC	TreatC	*p* value
ACE	576.209 ± 3.967	563.702 ± 6.409	0.134	574.616 ± 7.046	580.255 ± 3.650	0.474	570.129 ± 7.981	571.876 ± 5.592	0.86
Chao1	587.885 ± 6.454	574.586 ± 9.782	0.295	581.389 ± 8.257	582.350 ± 3.8100	0.914	577.426 ± 8.091	583.689 ± 7.49	0.576
Shannon	3.365 ± 0.090	3.704 ± 0.102	0.025	3.541 ± 0.068	3.8530 ± 0.103	0.023	3.510 ± 0.166	3.780 ± 0.112	0.193
Simpson	0.109 ± 0.015	0.070 ± 0.008	0.026	0.096 ± 0.008	0.072 ± 0.009	0.071	0.121 ± 0.023	0.077 ± 0.011	0.108

## Data Availability

The data sets of this study are available on request to the corresponding author.

## Supplementary Materials

Supplementary Materials Table S1. Differential metabolites of TreatA rat between ConA rat. Table S2. Differential metabolites of TreatC rat between ConC rat.
