# Supplementary material for: Effects of Herbal Therapy on Intestinal Microbiota and Serum Metabolomics in Different Rat Models of Mongolian Medicine
Source: Evid Based Complement Alternat Med. 2022 May 30;2022:7255780. doi: 10.1155/2022/7255780 (PMC9170395; doi:10.1155/2022/7255780)
Supplement: Supplementary Materials — Table S1. Differential metabolites of TreatA rat between ConA rat. Table S2. Differential metabolites of TreatC rat between ConC rat. [file 7255780.f1.zip › 7255780.f1/Table S1.doc]

Table S1 Differential metabolites of TreatA rat between ConA rat

| **NO** | **Name** | **Formula** | **RT [min]** | **Mode** | **VIP** | **P** |
| --- | --- | --- | --- | --- | --- | --- |
| 1 | Phthalic acid | C8 H6 O4 | 12.507 | Positiveion | 1.26652 | 0.045151502 |
| 2 | PEG n10 | C20 H42 O11 | 4.769 | Positiveion | 1.45019 | 0.030269773 |
| 3 | Istamycin C1 | C19 H37 N5 O6 | 4.435 | Positiveion | 1.35849 | 0.042143558 |
| 4 | PEG n11 | C22 H46 O12 | 4.871 | Positiveion | 1.37283 | 0.042808807 |
| 5 | Creatinine | C4 H7 N3 O | 0.802 | Positiveion | 1.23659 | 0.030859632 |
| 6 | LysoPC(22:4(7Z,10Z,13Z,16Z)) | C30 H54 N O7 P | 10.044 | Positiveion | 1.51987 | 0.005786545 |
| 7 | (2R)-2-Acetoxy-3-[(9Z)-9-octadecen-1-yloxy]propyl 2-(trimethylammonio)ethyl phosphate | C28 H56 N O7 P | 10.035 | Positiveion | 1.55221 | 0.004455589 |
| 8 | LysoPC(22:5(7Z,10Z,13Z,16Z,19Z)) | C30 H52 N O7 P | 9.403 | Positiveion | 1.2911 | 0.039363958 |
| 9 | PEG n12 | C24 H50 O13 | 4.764 | Positiveion | 1.43192 | 0.023706558 |
| 10 | 1-stearoyl-sn-glycero-3-phosphoethanolamine | C23 H48 N O7 P | 9.227 | Positiveion | 1.49851 | 0.00997637 |
| 11 | PEG n6 | C12 H26 O7 | 4.28 | Positiveion | 1.41777 | 0.032498713 |
| 12 | PEG n13 | C26 H54 O14 | 5.015 | Positiveion | 1.37127 | 0.040206787 |
| 13 | Victoria Pure Blue BO | C33 H39 N3 | 9.046 | Positiveion | 1.21996 | 0.028814823 |
| 14 | Glycerophospho-N-palmitoyl ethanolamine | C21 H44 N O7 P | 9.315 | Positiveion | 1.99491 | 0.00019244 |
| 15 | 1-(1Z-hexadecenyl)-sn-glycero-3-phosphocholine | C24 H50 N O6 P | 10.605 | Positiveion | 1.29978 | 0.024254388 |
| 16 | Cholinephosphate | C5 H14 N O4 P | 9.109 | Positiveion | 1.43613 | 0.019818754 |
| 17 | 1,3-di-o-Tolylguanidine | C15 H17 N3 | 5.433 | Positiveion | 1.14608 | 0.040014283 |
| 18 | PEG-4 | C8 H18 O5 | 3.932 | Positiveion | 1.36915 | 0.043698246 |
| 19 | Gentian violet | C25 H29 N3 | 7.754 | Positiveion | 2.06466 | 9.16E-06 |
| 20 | Cytidine | C9 H13 N3 O5 | 0.902 | Positiveion | 1.82615 | 0.000398994 |
| 21 | 3138020 | C15 H22 O2 | 6.143 | Positiveion | 1.80751 | 0.000651216 |
| 22 | 9-Decenoylcarnitine | C17 H31 N O4 | 6.706 | Positiveion | 1.38897 | 0.021013345 |
| 23 | tetrahydrothiophene1-oxide | C4 H8 O S | 0.913 | Positiveion | 2.25303 | 1.62E-07 |
| 24 | 6-Methoxyquinoline | C10 H9 N O | 4.433 | Positiveion | 1.48763 | 0.005198972 |
| 25 | Taurine | C2 H7 N O3 S | 0.764 | Positiveion | 1.35481 | 0.035905459 |
| 26 | Melatonin | C13 H16 N2 O2 | 5.203 | Positiveion | 1.51422 | 0.008020326 |
| 27 | 4-Coumarylalcohol(4-Hydroxycoumarin) | C9 H6 O3 | 4.772 | Negativeion | 2.12537 | 1.63E-05 |
| 28 | 4-vinylguaiacol sulfate | C9 H10 O5 S | 6.154 | Negativeion | 1.87579 | 0.00041054 |
| 29 | 3-oxopalmitic acid | C16 H30 O3 | 9.63 | Negativeion | 1.37209 | 0.045919506 |
| 30 | Palmitoleicacid | C16 H30 O2 | 10.675 | Negativeion | 1.73494 | 0.008303297 |
| 31 | Pseudouridine | C9 H12 N2 O6 | 0.903 | Negativeion | 1.5819 | 0.010830979 |
| 32 | Hexadecanoicacid | C16 H32 O2 | 11.383 | Negativeion | 1.85623 | 0.001450476 |
| 33 | 3-(7-Hydroxy-4-oxo-4H-chromen-2-yl)phenyl hydrogen sulfate | C15 H10 O7 S | 5.515 | Negativeion | 1.30854 | 0.027474425 |
| 34 | 2646 | C15 H26 O2 | 7.772 | Negativeion | 1.53985 | 0.030602702 |
| 35 | 1-hexadecanoyl-sn-glycero-3-phosphoethanolamine | C21 H44 N O7 P | 9.309 | Negativeion | 1.5065 | 0.034428609 |
| 36 | 4-vinylphenol sulfate | C8 H8 O4 S | 5.756 | Negativeion | 1.46109 | 0.012489624 |
| 37 | [FA(18:2)]9Z_11E-octadecadienoicacid | C18 H32 O2 | 10.841 | Negativeion | 1.47465 | 0.023365496 |
| 38 | (9Z)-(13S)-12_13-Epoxyoctadeca-9_11-dienoicacid | C18 H30 O3 | 9.492 | Negativeion | 1.39708 | 0.045239888 |
| 39 | (R)-10-Hydroxystearate | C18 H36 O3 | 11.028 | Negativeion | 1.97036 | 0.002015216 |
| 40 | callystatin A | C29 H44 O4 | 9.026 | Negativeion | 1.22448 | 0.040170634 |
| 41 | (6Z)-Octadecenoicacid | C18 H34 O2 | 11.502 | Negativeion | 1.72968 | 0.004654008 |
| 42 | 4-Ethyl-2-methoxyphenyl hydrogen sulfate | C9 H12 O5 S | 6.488 | Negativeion | 1.68485 | 0.002027895 |
| 43 | 1-(4-Methoxyphenyl)-4-methyl-3-oxopentyl hydrogen sulfate | C13 H18 O6 S | 6.407 | Negativeion | 1.23151 | 0.040657153 |
| 44 | 2-Oxooctadecanoicacid | C18 H34 O3 | 10.368 | Negativeion | 1.59239 | 0.021325443 |
| 45 | Ubiquinone Q4 | C29 H42 O4 | 9.544 | Negativeion | 1.27718 | 0.039426831 |
