# Supplementary material for: Effects of Herbal Therapy on Intestinal Microbiota and Serum Metabolomics in Different Rat Models of Mongolian Medicine
Source: Evid Based Complement Alternat Med. 2022 May 30;2022:7255780. doi: 10.1155/2022/7255780 (PMC9170395; doi:10.1155/2022/7255780)
Supplement: Supplementary Materials — Table S1. Differential metabolites of TreatA rat between ConA rat. Table S2. Differential metabolites of TreatC rat between ConC rat. [file 7255780.f1.zip › 7255780.f1/Table S2.doc]

Table S2 Differential metabolites of TreatC rat between ConC rat

| **NO** | **Name** | **Formula** | **RT [min]** | **Mode** | **VIP** | **P** |
| --- | --- | --- | --- | --- | --- | --- |
| 1 | 1-Oleoylglycerophosphocholine | C26 H52 N O7 P | 10.106 | Positiveion | 1.34006 | 0.007804166 |
| 2 | Bis(2-ethylhexyl) phthalate | C24 H38 O4 | 12.508 | Positiveion | 1.12595 | 0.011914257 |
| 3 | Erucamide | C22 H43 N O | 13.314 | Positiveion | 1.37939 | 0.006252725 |
| 4 | Choline | C5 H13 N O | 0.773 | Positiveion | 1.22248 | 0.022688444 |
| 5 | Phthalic acid | C8 H6 O4 | 12.507 | Positiveion | 1.12109 | 0.012799103 |
| 6 | PEG n8 | C16 H34 O9 | 4.277 | Positiveion | 1.2518 | 0.023779782 |
| 7 | PEG n10 | C20 H42 O11 | 4.558 | Positiveion | 1.32361 | 0.012441648 |
| 8 | Istamycin C1 | C19 H37 N5 O6 | 4.435 | Positiveion | 1.46961 | 0.00629335 |
| 9 | Palmitoylcarnitine | C23 H45 N O4 | 9.641 | Positiveion | 1.43244 | 0.003714294 |
| 10 | PEG n12 | C24 H50 O13 | 4.776 | Positiveion | 1.4045 | 0.005838914 |
| 11 | PEG n13 | C26 H54 O14 | 5.027 | Positiveion | 1.26365 | 0.022505823 |
| 12 | PEG n14 | C28 H58 O15 | 5.102 | Positiveion | 1.27557 | 0.019687616 |
| 13 | PEG n16 | C32 H66 O17 | 5.233 | Positiveion | 1.24969 | 0.017498389 |
| 14 | PEG n15 | C30 H62 O16 | 5.023 | Positiveion | 1.21736 | 0.027127292 |
| 15 | stearoylcarnitine | C25 H49 N O4 | 10.843 | Positiveion | 1.0534 | 0.039324285 |
| 16 | L-alpha-lysophosphatidylcholine | C22 H46 N O7 P | 8.711 | Positiveion | 1.25993 | 0.023472945 |
| 17 | Ectoine | C6 H10 N2 O2 | 0.85 | Positiveion | 1.0466 | 0.036842386 |
| 18 | Glycerophospho-N-palmitoyl ethanolamine | C21 H44 N O7 P | 9.315 | Positiveion | 1.70121 | 3.86E-05 |
| 19 | Eicosapentaenoic acid | C20 H30 O2 | 9.322 | Positiveion | 1.57974 | 0.00026281 |
| 20 | 2-linoleoyl-sn-glycero-3-phosphoethanolamine | C23 H44 N O7 P | 8.945 | Positiveion | 1.30059 | 0.008911466 |
| 21 | D-Tryptophan | C11 H12 N2 O2 | 4.429 | Positiveion | 1.06654 | 0.035982872 |
| 22 | DL-Stachydrine | C7 H13 N O2 | 0.871 | Positiveion | 1.23898 | 0.024534821 |
| 23 | (2R)-1-{[(2-Aminoethoxy)(hydroxy)phosphoryl]oxy}-3-hydroxy-2-propanyl (11Z)-11-icosenoate | C25 H50 N O7 P | 9.486 | Positiveion | 1.32379 | 0.005662467 |
| 24 | Cholinephosphate | C5 H14 N O4 P | 9.109 | Positiveion | 1.49687 | 0.001123695 |
| 25 | 1-oleoyl-sn-glycero-3-phosphoethanolamine | C23 H46 N O7 P | 9.52 | Positiveion | 1.60078 | 0.000712658 |
| 26 | 2-ETHYL-4,5-DIMETHYLOXAZOLE | C7 H11 N O | 0.901 | Positiveion | 1.26184 | 0.016555364 |
| 27 | 1-Hexadecanoylpyrrolidine | C20 H39 N O | 11.891 | Positiveion | 1.30585 | 0.013031043 |
| 28 | tranexamic acid | C8 H15 N O2 | 0.893 | Positiveion | 1.1354 | 0.023963179 |
| 29 | LQ1825000 | C7 H13 N O | 0.892 | Positiveion | 1.28739 | 0.018889226 |
| 30 | RG5927903 | C13 H22 O2 | 11.329 | Positiveion | 1.09726 | 0.046355019 |
| 31 | Pregabalin | C8 H17 N O2 | 0.892 | Positiveion | 1.36239 | 0.009705202 |
| 32 | Taurocholic acid | C26 H45 N O7 S | 6.011 | Positiveion | 1.0979 | 0.035613376 |
| 33 | Capsidiol | C15 H24 O2 | 6.596 | Positiveion | 1.1996 | 0.014664211 |
| 34 | Nootkatone | C15 H22 O | 6.604 | Positiveion | 1.20823 | 0.013859074 |
| 35 | 15-Acetyldeoxynivalenol | C17 H22 O7 | 5.51 | Positiveion | 1.0771 | 0.032642863 |
| 36 | D-Sphingosine | C18 H37 N O2 | 8.478 | Positiveion | 1.6373 | 0.000138932 |
| 37 | Taurine | C2 H7 N O3 S | 0.764 | Positiveion | 1.21375 | 0.016918409 |
| 38 | Oleamide | C18 H35 N O | 8.5 | Positiveion | 1.52978 | 0.000555922 |
| 39 | Isopropyl methoxy pyrazine | C8 H12 N2 O | 0.906 | Positiveion | 1.36411 | 0.003626179 |
| 40 | (5Z_13E)-6_9alpha-Epoxy-11alpha-hydroxy-15-oxoprosta-5_13-dienoate | C20 H30 O5 | 8.418 | Negativeion | 2.13438 | 5.15E-05 |
| 41 | 4-vinylguaiacol sulfate | C9 H10 O5 S | 6.154 | Negativeion | 1.48108 | 0.026553756 |
| 42 | Cyclic-3,20-bis(1,2-ethanediyl acetal)-11alpha-(acetyloxy)-5alpha,6alpha-epoxypregnane-3,20-dione | C27 H40 O7 | 7.379 | Negativeion | 1.29867 | 0.045261067 |
| 43 | D-erythro-Sphingosine1-phosphate | C18 H38 N O5 P | 8.493 | Negativeion | 1.88003 | 0.002001814 |
| 44 | 3alpha_12alpha-Dihydroxy-7-oxo-5beta-cholanate | C24 H38 O5 | 6.957 | Negativeion | 1.57377 | 0.008749999 |
| 45 | GibberellinA12 | C20 H28 O4 | 8.417 | Negativeion | 2.10495 | 7.66E-05 |
| 46 | 13-Hydroxy-9-methoxy-10-oxo-11-octadecenoic acid | C19 H34 O5 | 9.584 | Negativeion | 1.57776 | 0.020022087 |
| 47 | 2-(3,4-Dihydroxyphenyl)-3,7-dihydroxy-5-chromanesulfinic acid | C15 H14 O7 S | 5.948 | Negativeion | 1.54469 | 0.020976987 |
| 48 | Taxa-4(20)_11(12)-dien-5alpha-acetoxy-10beta-ol | C22 H34 O3 | 9.606 | Negativeion | 1.73139 | 0.011783003 |
| 49 | (5Z_9E_14Z)-(8xi_11R_12S)-11_12-Epoxy-8-hydroxyicosa-5_9_14-trienoicacid | C20 H32 O4 | 8.745 | Negativeion | 1.85724 | 0.001160156 |
| 50 | Medroxyprogesterone | C22 H32 O3 | 9.217 | Negativeion | 1.53301 | 0.028642695 |
| 51 | [FA(18:0)]12R_13S-epoxy-9Z-octadecenoicacid | C18 H32 O3 | 9.037 | Negativeion | 1.53812 | 0.041632786 |
| 52 | guaiacol sulfate | C7 H8 O5 S | 4.66 | Negativeion | 1.88389 | 0.000522998 |
| 53 | MFCD12546417 | C6 H6 O5 S | 4.175 | Negativeion | 1.94791 | 0.000312129 |
| 54 | [FA(20:3)]11_12-epoxy-5Z_8Z_14Z-eicosatrienoicacid | C20 H32 O3 | 9.306 | Negativeion | 1.735 | 0.006625359 |
| 55 | 3alpha_7alpha_12beta-Trihydroxy-5beta-cholanate | C24 H40 O5 | 7.379 | Negativeion | 1.43488 | 0.019105144 |
| 56 | anacardic acid | C22 H36 O3 | 9.783 | Negativeion | 1.47782 | 0.040262191 |
| 57 | 3-Ureidoisobutyrate | C5 H10 N2 O3 | 0.781 | Negativeion | 1.45778 | 0.040705707 |
| 58 | 3138020 | C15 H22 O2 | 9.066 | Negativeion | 1.51013 | 0.014429576 |
| 59 | alpha-L-Arabinose | C5 H10 O5 | 0.806 | Negativeion | 1.42543 | 0.049310166 |
| 60 | 2-Carboxy-D-arabinitol | C6 H12 O7 | 0.798 | Negativeion | 1.43677 | 0.040721887 |
| 61 | [FA(20:4)]17R_18S-epoxy-5Z_8Z_11Z_14Z-eicosatetraenoicacid | C20 H30 O3 | 8.897 | Negativeion | 1.72613 | 0.006141294 |
| 62 | 15S-hydroxyeicosatrienoic acid | C20 H34 O3 | 9.493 | Negativeion | 2.16126 | 9.04E-05 |
